# High-Resolution Sonography: A New Technique to Detect Nerve Damage in Leprosy

**DOI:** 10.1371/journal.pntd.0000498

**Published:** 2009-08-11

**Authors:** Suman Jain, Leo H. Visser, T. L. N. Praveen, P. Narasimha Rao, Thummalakunta Surekha, Ramesh Ellanti, Thummalakunta L. N. Abhishek, Indira Nath

**Affiliations:** 1 Clinical and Epidemiology Division, Blue Peter Research Centre, LEPRA Society, Cherlapally, Hyderabad, India; 2 Department of Neurology and Clinical Neurophysiology, St. Elisabeth Hospital, Tilburg, The Netherlands; 3 Abhishek Institute of Imageology, Secunderabad, India; Kwame Nkrumah University of Science and Technology (KNUST) School of Medical Sciences, Ghana

## Abstract

**Background:**

Leprosy is the most common treatable peripheral nerve disorder worldwide with periods of acute neuritis leading to functional impairment of limbs, ulcer formation and stigmatizing deformities. Since the hallmarks of leprosy are nerve enlargement and inflammation, we used high-resolution sonography (US) and color Doppler (CD) imaging to demonstrate nerve enlargement and inflammation.

**Methology/Principal Findings:**

We performed bilateral US of the ulnar (UN), median (MN), lateral popliteal (LP) and posterior tibial (PT) nerves in 20 leprosy patients and compared this with the clinical findings in these patients and with the sonographic findings in 30 healthy Indian controls.

The nerves were significantly thicker in the leprosy patients as compared to healthy controls (p<0.0001 for each nerve). The two patients without nerve enlargements did not have a type 1 or type 2 reaction or signs of neuritis. The kappa for clinical palpation and nerve enlargement by sonography was 0.30 for all examined nerves (0.32 for UN, 0.41 for PN and 0.13 for LP). Increased neural vascularity by CD imaging was present in 39 of 152 examined nerves (26%). Increased vascularity was observed in multiple nerves in 6 of 12 patients with type 1 reaction and in 3 of 4 patients with type 2 reaction. Significant correlation was observed between clinical parameters of grade of thickening, sensory loss and muscle weakness and US abnormalities of nerve echotexture, endoneural flow and cross-sectional area (p<0.001).

**Conclusions/Significance:**

We conclude that clinical examination of enlarged nerves in leprosy patients is subjective and inaccurate, whereas sonography provides an objective measure of nerve damage by showing increased vascularity, distorted echotexture and enlargement. This damage is sonographically more extensive and includes more nerves than clinically expected.

## Author Summary

*Mycobacterium leprae*, which causes leprosy, infects peripheral nerves resulting in functional impairment, ulcer formation and stigmatizing deformities. Early diagnosis of nerve involvement is important to avoid nerve related complications. We used non-invasive, high-resolution sonography (US) and color Doppler (CD) imaging to study the ulnar (UN), median (MN), lateral popliteal (LP) and posterior tibial (PT) nerves in 20 leprosy patients and compared 30 healthy Indian controls. The nerves were significantly thicker in the patients (p<0.0001 for each nerve). One of the key signs of leprosy is the presence of enlarged nerves. The kappa for clinical palpation and nerve enlargement by sonography was 0.30 for all examined nerves. Increased neural vascularity, the sign of inflammation was observed in 26% (39/152) of nerves by CD imaging. Increased CD was observed in multiple nerves in 3 of 4 patients with type 2 reaction. Significant correlation was observed between clinical parameters of grade of thickening, sensory loss and muscle weakness and US abnormalities of nerve echotexture, endoneural flow and cross-sectional area (p<0.001). We conclude that sonography is a better diagnostic tool to predict nerve damage as compared to clinical assessment. Nerve damage was sonographically more extensive and was observed in nerves considered clinically normal.

## Introduction

Leprosy is the most common treatable peripheral nerve disorder worldwide [1]. Leprosy is caused by a chronic granulomatous immune response to infection of the skin and nerves with *Mycobacterium leprae*, which resides in macrophages and Schwann cells and is the only bacterium known to affect myelination and cause peripheral neuropathy. Nerve damage, affecting mainly the ulnar (UN), median (MN), and posterior tibial (PT) nerves, results in nerve enlargement.

Leprosy presents as a clinico-pathological spectrum [2] ranging from the localized paucibacillary tuberculoid form with anaesthetic hypopigmented skin patch (TT) to the generalized multibacillary, lepromatous leprosy. Between these poles are unstable forms of borderline tuberculoid, borderline borderline and borderline lepromatous leprosy. These are prone to episodic exacerbations (reactions) in 15–50% of patients during the course of the disease and after the completion of multidrug therapy. These states include type 1 (reversal reaction), where only the skin patch shows inflammation with tenderness in the associated nerve, and type 2 [Erythema Nodosum Leprosum (ENL)] reaction, manifesting with systemic symptoms of fever, erythematous nodules and joint pains. Though some nerve involvement may be seen in all types of leprosy, leprosy reactions lead to severe morbidity and acute neuritis requiring immediate treatment. Efforts to diagnose early (or subclinical) neuritis could ameliorate the nerve damage leading to functional impairment of limbs, ulcer formation and stigmatizing deformities. Hence, the most important goal in the management of leprosy is the prevention of disability via early detection of nerve impairment [1].

Careful clinical testing is useful, but can only detect the presence of neuropathy. However if neuropathy is found, there already is a substantial amount of nerve damage [1]. Nerve conduction studies or warm perception testing may improve early detection strategies, but these are usually not available in leprosy centers [1]. Since the hallmarks of leprosy are nerve enlargement and inflammation, we decided to use high-resolution sonography to demonstrate nerve enlargement (even subclinically) and inflammation. Inflammation can be detected by increased blood flow signals in the epi- and endoneurium of the involved nerves in leprosy patients [3].

Therefore, we performed an extensive sonographic study in 20 leprosy patients and compared the sonographic findings with 30 healthy Indian controls.

## Materials and Methods

### Subjects

Thirty healthy volunteers 15 of each gender, aged between 17 to 58 years (mean 33±10) without any evidence of diabetes, hypothyroidism, HIV and trauma-related peripheral nerve disease were included in the study to obtain normal values of the cross-sectional areas (CSAs) of the MN, UN, lateral popliteal (LP) and PT nerves. Twenty leprosy patients, diagnosed as per Ridley-Jopling classification [2], who were in different stages of therapy with WHO multi-drug therapy [4], were included for evaluation. The study was approved by Blue Peter Research Centre Ethics Committee during 6th IEC held on19th December 2007. All the subjects were included in the study only after obtaining an informed written consent.

### Clinical evaluation/grading of nerves

All the volunteers and patients were examined by two clinicians trained in leprosy to assess bilaterally the UN, MN, LP and PT nerves. All the nerves were examined for their motor and sensory functions as follows.

1. UN: Each patient was screened for current symptoms of lesions of the ulnar nerve, i.e. numbness and paraesthesias of the fourth and fifth digits of the hand, medial elbow pain, weakness or clumsiness of the hand muscles innervated by the ulnar nerve. Both arms were examined by testing (1) pin-prick sensation at digit 5 using monofilaments, and (2) strength of the first dorsal interosseous (FDI) and abductor digiti minimi (ADM) using the Medical Research Council (MRC) rating scale [5].
2. MN: We evaluated pin-prick sensation in the distribution of the median nerve using monofilaments and assessment of motor function of abductor pollicis brevis (APB).
3. LP: The strength of the extensor hallucis longus and M. tibial anterior was tested using the Medical Research Council (MRC) rating scale
4. PT: Current symptoms of lesion of the posterior tibial nerve were tested by pin-prick sensation at the heel and sole of the foot using monofilaments and the muscle strength of the toe and foot flexors.

Sensory testing used Semmes-Weinstein monofilaments (SW) as previously described [6],[7]. Sensory loss was considered to be present when the patient was unable to perceive 2 grams of target force on the hand and 300 grams target force on the foot by SW filaments. Muscle weakness was present when the MRC score was 4 or less.

UN, LP and PTN were clinically graded after palpation as follows. Grade 0 was defined as a nerve not thicker than the contralateral nerve and with normal sensation; Grade 1 occurred when the affected nerve was thicker than the contralateral nerve; Grade 2 was a thickening of the affected nerve which felt rope-like; Grade 3 was a thickened nerve which felt beaded or nodular. Clinical grading of nerve thickening based on palpation could not be performed on MN due to its deeper location.

Skin smears were taken from three sites for presence of acid-fast bacilli and to assess the Bacillary Index (BI). Skin biopsy was performed to confirm the clinical diagnosis.

### Ultrasonography (US) and color doppler (CD)

All peripheral nerves were imaged by an independent radiographer blinded to the clinical diagnosis using US (Voluson -730 Expert, GE medical, USA) with broadband frequency of 10–14 MHz; CD frequency of 6–13 MHz and linear array transducer. Bilaterally, the MN at the wrist and forearm, the UN at the elbow and proximal to the medial epicondyle, LP at the fibula head and PT nerves at the ankle and proximal to the medial malleolus were examined and the length of abnormality of the nerve was determined by the presence of abnormal size and echo reflectivity of the nerves. All nerves were measured on transverse sections at a point where the nerve thickness was maximum in the visualized segment of the nerve. On transverse scans, the cross-sectional area of the nerve was determined from that area by one measurement within the hyperechoic rim surrounding the nerve.

The echo reflectivity of the nerves assessed on imaging was arbitrarily graded as follows: mild = some hypo-reflectivity, moderate = obvious hypo-reflectivity; and severe = absence of any fascicular pattern.

Color Doppler (CD) settings were chosen to optimize identification of weak signals from vessels with slow velocity. Pulse repetition frequency was set of 1 KHZ and Doppler gain was adjusted to the maximum level that thus not produce clutter. Band filter was set at 50 Hz. The presence of blood flow signals in the perineural plexus or intrafascicular vessels indicated hypervascularity of the nerve during CD imaging

### Statistical analysis

Statistical analysis was performed using SPSS software version 11/ graph pad prism version 4.

For comparison of group differences, the one-way nonparametric analysis of variance (Kruskal-Wallis test) or the Wilcoxon-Mann-Whitney test were used. For the comparison of proportions the χ^2^ test was used. Probability (p) values less than 0.05 were considered significant.

## Results

### Healthy subjects

**Figure 1:**
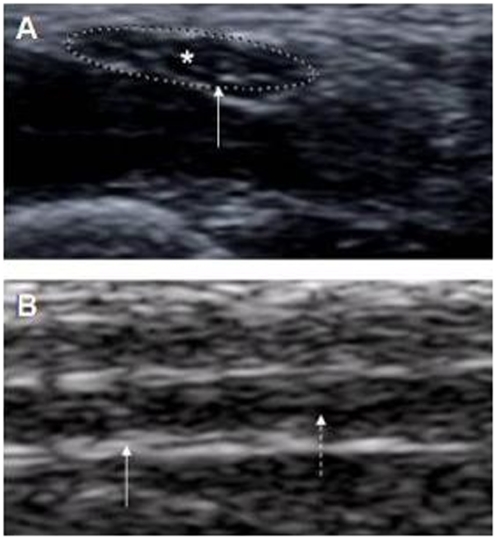
Ultrasonography and color Doppler images of peripheral nerves of healthy subjects. A Transverse scan of medial nerve from a healthy subject as denoted by dotted ellipse (CSA = 4.5 mm^2^) showing hypoechoic fascicles separated by hyperechoic areas in a ‘honeycomb’ like pattern with absence of blood flow signals; B longitudinal ultra sonogram of ulnar nerve from a healthy subject (arrows) with hyperechogenic bands in a linear pattern appearing as bundles of straw.

Thirty ulnar, median, and posterior tibial, and 23 lateral popliteal nerves (not all female volunteers allowed the LP nerve to be examined due to cultural/social reasons) were examined. On palpation, all the nerve trunks were of normal size (grade 0) and not tender. On US, the peripheral nerves appeared as round to oval with occasional internal punctuate echoes giving a ‘honey comb pattern’ in transverse scans (Fig. 1A), and as hypoechoic tubular structures with parallel linear internal echoes suggestive of ‘bundles of straw’ in longitudinal scans (Fig. 1B). The epi- and perineurium were uniformly hyperechoic with an absence of endo- and epineural blood flow signals on CD imaging. The mean CSA for all 4 nerves showed no age or gender related differences (p>0.1). The ulnar nerve showed the highest mean CSA as compared to the other nerves (table 1).

**Table 1:** Cross sectional area (mm^2^) of major peripheral nerve trunks of upper and lower limbs of healthy subjects and leprosy patients.

Subjects	Ulnar nerves (30)	Median nerves (30)	Lateral popliteal nerves (23)	Posterior tibial nerves (30)
**Healthy subjects (30)**				
Mean±SD	8.5±3.5	6.2±2.2	5.9±3.2	6.3±3.2
Median	7.7	6	6.1	5.6
Range	3–16.9	3.23–12.2	3–12.	2–15.7
**Leprosy patients (20)**	(39)	(39)	(38)	(36)
Mean±SD	22.7±19.4	14.7±11.6	12.8±7.	12.±8.
Median	14.4	11.6	11.6	8.8
Range	4.6–89.8	4–69.1	4–38	2–36.8
p-Value	<0.0001	<0.0001	<0.0001	<0.0001

Figures in parenthesis indicate number of subjects. p value by Mann-Whitney test.

### Leprosy patients

#### General characteristics

The profile of the 20 age-matched leprosy patients included in the study is provided in Table 2. The duration of disease ranged from 3 to 180 (mean 24.7±39.8) months. Ten patients had borderline tuberculoid, 3 borderline lepromatous and 7 lepromatous leprosy. Twelve had type 1 reaction and 4 patients had type 2 reactions, which was associated with neuritis. Four patients (2 borderline tuberculoid and 2 lepromatous leprosy) had no clinical evidence of reaction or neuritis. Skin smears were positive in 10 patients. Clinical thickening, ranging from grade 1 to 3, was observed in 86 nerves of the 120 examined nerves (72%; table 3).

**Table 2:** Profile of leprosy patients.

Leprosy type	Age (years)	Sex		Duration of disease (months)	Doses of MDT (no.)	Skin Smear Positive (N)	Type of leprosy Reactions	
		Male	Female				Type 1	Type 2
Borderline tuberculoid (10)	12–52	9	1	1–180	1–11	0	8	0
Borderline lepromatous (3)	27–52	3	0	6–36	12–24	3	2	1
Lepromatous leprosy (7)	18–52	6	1	1–60	1–24	7	2	3

MDT = multidrug therapy; (N) = Number of patients.

**Table 3:** Clinical and sonographic findings of major peripheral nerves of upper and lower limbs in 20 leprosy patients.

Characteristics	Ulnar nerves (40)	Median nerves (40)	Lateral popliteal nerves (40)	Posterior tibial nerves (40)	All nerves(160)
**Clinical Involvement**					
Thickening					
Grade 0	7 (17.5%)		10 (25%)	17 (42.5%)	34 (28.3%)
Grade 1	20 (50%)		30 (75%)	23 (57.5%)	73 (60.8%
Grade 2	8 (20%)		0	0	8 (6.6%)
Grade 3	5 (12.5%)		0	0	5 (4.2%)
Sensory loss	11 (27.5%)	7 (17.5%)	6 (15%)	6 (15%)	30 (18.7%)
Motor weakness	17 (42.5%)	6 (15%)	1 (2.5%)	2 (5%)	26 (16.3%)
Both Motor and Sensory loss	10 (25%)	4 (10%)	0	2 (5%)	16 (10%)
Normal	2 (5%)	23 (57.5%)	33 (82.5%)	30 (75%)	88 (55%)
**Sonographic findings**	(39)	(39)	(38)	(36)	(152)
Echo reflectivity					
Normal	11 (28.2%)	18 (46.2%)	24 (63.2%)	23(63.9%)	76 (50%)
Mild	4 (10.3%)	5 (12.8%)	10 (26.3%)	6 (16.7%)	25 (16.4%)
Moderate	19 (48.7%)	15 (38.5%)	4 (10.5%)	7 (19.4%)	45 (29.7%)
Severe reduced	5 (12.8%)	1 (2.5)	0	0	6 (3.9%)
CSA Enlargement*	17(43.6%)	23(58.9%)	18 (47.3%)	12 (33.3%)	70 (46.1%)
Increased CD	23 (58.9%)	10 (25%)	4 (10.5%)	2 (5.5%)	39 (25.6%)

Clinical grading of thickness of Median nerve could not be done due to its deeper location under flexor retinaculum. Figures in parenthesis indicate number of nerves characteristics and sonographic findings rows and in other places it indicates percentages. CSA = cross-sectional area.

*: Based on more than Mean+2SD values in healthy subjects.

#### Clinical characteristics and sonography

**Figure 2:**
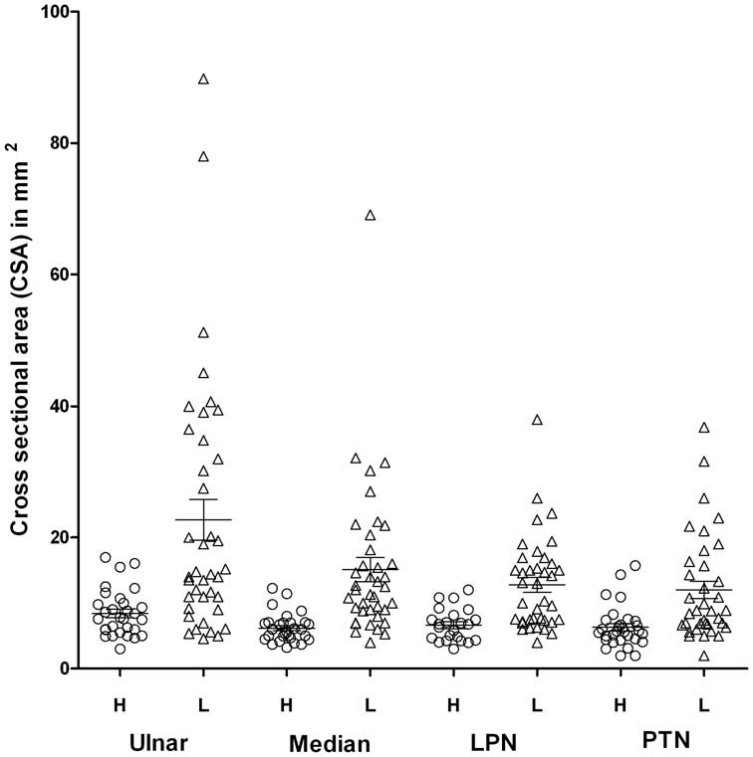
Comparison of mean cross-sectional areas (CSA in mm^2^) in nerves from leprosy patients as compared to healthy control subjects. The mean CSA in mm^2^±SD as determined by ultrasonography from 20 leprosy patients (L, open triangle) showed highly significant increase (p<0.0001, Mann Whitney) as compared to 30 healthy control subjects (H, open circle) for ulnar, median, lateral popliteal (LPN) and posterior tibial nerves (PTN). There was a wider scatter in the diseased nerves as compared to healthy controls.

Table 1 and Fig. 2 show the mean CSAs of the different nerves in leprosy patients versus controls. The nerves were significantly thicker in the leprosy patients as compared to controls (p<0.0001 for each nerve).

Eighteen of the 20 leprosy patients had one or more nerves which were enlarged (based on the upper limit of normal mean+2SD). The two patients who did not have nerve enlargements did not have a type 1 or type 2 reaction or signs of neuritis. Very enlarged nerves with a CSA>50 mm^2^ were observed in four nerves (3 UN and 1MN) and all these patients had a type 1 reaction.

When the sonographic findings and the clinical characteristics (table 3) were analysed, significant differences were observed in the mean CSA for clinical grades 0 versus grades 1 (p = 0.02), 2 (p = 0.002) and 3 (p = 0.0003). In the 34 nerves for which clinical thickening was not observed (7 UN, 10 LP, and 17 PTN) by palpation (grade 0), the CSA was above the upper limit of normal in 5 nerves (3 LP and 2 PT). On the contrary, 39 of the 86 clinically thickened nerves (33 UN, 30 LP, and 23 PT) did not show sonographic enlargement. Clinical grade 2 and higher nerve enlargements were only found for UN, but not for LP and PT nerves. UN did not show sonographic enlargement for any situation in which clinical thickening was not observed. The kappa of agreement between clinical palpation and nerve enlargement by sonography was 0.30 for all examined nerves (κ = 0.32 for UN, κ = 0.41 for PN and κ = 0.13 for LP).

CD flow signals were observed in 3 of the 34 clinically non-thickened nerves (8%), two of them in UN and one in LP. Blood flow signals were observed in 17 of 73 grade 1- thickened nerves (23%), 4 of 8 grade 2-thickened nerves (50%) and 5 of 5 grade 3- thickened nerves (100%). This indicated that the more the nerve was clinically enlarged, the more often CD flow signals were present (p<0.0001, table 4)

**Table 4:** Statistical significance (p value) of the relationship of clinical parameters versus ultrasonographic features in 152 peripheral nerves of leprosy patients.

Clinical Parameters	Ultrasonographic Features		
	CSA	Echotexture	Endo/perineural Blood flow signal
Clinical grading of nerve thickening (120)	0.0001*	0.0001	0.0001
Presence of sensory loss (152)	0.011*	0.001	0.002
Presence of muscle weakness(152)	0.001*	0.004	0.026

*: Kruskal-Wallis test, otherwise χ^2^.

In the 30 nerves of 12 patients with sensory loss (table 3), the nerve supplying the area of sensory loss was sonographically enlarged in 23 nerves (77%). In the 26 nerves of 15 patients presenting with motor weakness, thickening was observed sonographically in 17 nerves (65%) and in the 16 nerves of 9 patients with both motor and sensory loss, 13 nerves (81%) were sonographically enlarged. Significant correlation was observed between clinical parameters of grade of thickening, sensory loss and muscle weakness and US abnormalities of CSA, echotexture, endoneural flow (p<0.001, table 4). CD flow signals was observed most frequently in the UN (23 of 39 nerves). Combined sensory and motor loss was observed in 7 of the 23 ulnar nerves with increased CD, only motor loss in 3 and only sensory loss in 1 nerve. Thus, in 12 of 23 nerves with CD flow signals, no sensory or motor impairment was observed.

#### Sonographic characteristics: echotexture and CD flow

Of the 152 nerves examined in the leprosy patients, 50% of the nerves showed normal echo patterns. In the remainder, moderate or severe reduced echo reflectivity, indicative of partial to total loss of fascicles structure, was seen over 2–22 cm length of the nerves. The longest lengths were measured in the UN and MN. UN was found to be most enlarged 4–6 cm above the sulcus and with MN approximately 4 cm proximal to carpal tunnel inlet. Mild, moderate and severe echo reflectivity changes were observed respectively in 16, 29% and 4% of the nerves examined (table 3). There was a highly significant correlation between CSA and echotexture grading (p = 0.0001).

**Figure 3:**
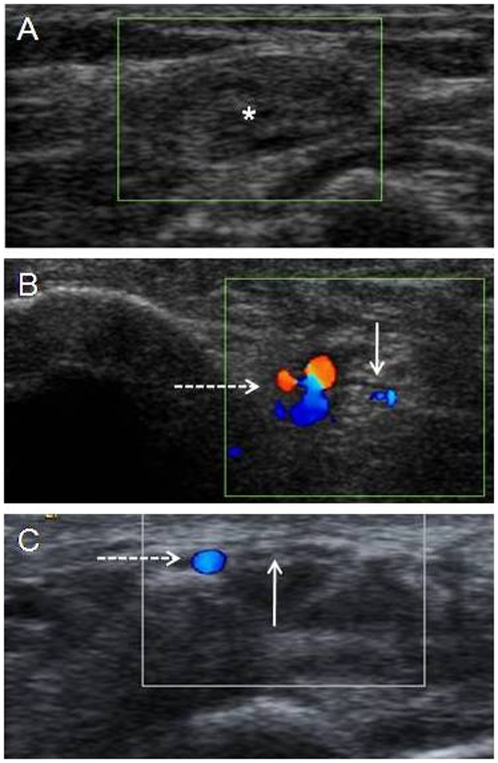
Ultrasonography and color Doppler images of peripheral nerves of leprosy patients. A transverse scan of an enlarged ulnar nerve indicated by an asterisk (CSA = 45 mm^2^) from BT leprosy patient with type 1 reaction showing no endoneural blood flow signals; B colour Doppler image of a right ulnar nerve in a BT patient (CSA = 51 mm^2^) undergoing type 1 leprosy reaction, showing increased endoneural and perineural blood flow signals (demarcated by solid arrow in a rectangular box, dashed arrow in a box shows a blood vessels) suggestive of acute neuritis; C colour Doppler image of a median nerve (shown by solid arrow) in a BT patient (CSA = 9 mm^2^) undergoing type 1 leprosy reaction with persistent artery (shown by dashed arrow), showing no endoneural flow signal.

Endo or perineural flow suggestive of increased neural vascularity by CD imaging was observed in 39 out of 152 examined nerves (26%). Neural vascularity was observed more frequently in upper limb nerves with UN being most affected (58%) followed by the median nerve (25%). Bilateral involvement was also seen more in the UN and the MN. In the lower limb nerves, LP (11%) showed a higher percentage of neural vascularity compared to PT nerves (6%). All the nerves with neural vascularity were from the 16 patients who had associated leprosy reactions. Fig. 3 and Video S1 show an example of increased flow in the UN (Fig. 3B) in comparison to UN without flow (Fig. 3A) and Fig. 3C shows no flow in MN in spite of a persisting artery. Of the 39 nerves with increased flow, 26 were from 12 patients with type 1 reaction and 13 were from the 4 patients with a type 2 reaction.

In 9 out of the 16 patients with associated leprosy reactions, neural vascularity on CD imaging were seen in the nerve trunk on the side of the inflammed skin lesions and in contralateral and distant nerve trunks, suggesting more extensive involvement of nerves during reactions of leprosy compared to clinical skin involvement. Increased CD was observed in multiple nerves in 6 out of 12 patients undergoing type 1 reaction, which is considered to be localized to the dermal lesions and the neighboring nerves. In patients with a type 2 reaction, blood flow signals in multiple nerves was seen in 3 out of 4 patients.

## Discussion

This study demonstrates the usefulness of US in detecting nerve damage in leprosy. Our findings may have clinical and therapeutical consequences. Peripheral nerves are often enlarged in leprosy, and these are more accurately assessed by US than by clinical palpation. UN is the most commonly involved nerve. Nerve enlargement is more often present in patients with type 1 or type 2 reaction and the nerves of these patients often showed an increased vascularity in both the clinically involved nerves and in nerves far distant from those clinically affected.

There is a growing interest in US as a diagnostic tool for diseases of the peripheral nervous system including mononeuropathies, polyneuropathies and peripheral nerve tumors [8]–[12]. US is noninvasive, amenable to studying structural changes in nerve sites that cannot be biopsied for histopathology, and is more cost effective than magnetic resonance imaging. Moreover, with US the nerve can be probed for a longer length than MRI examination which is limited to defined segments. Technical developments leading to improved image quality and reduced sizes of US equipment together with a reduction in price will make it possible for US to become a tool that can be used in countries where leprosy is still endemic.

This is the second study that shows the value of US and CD as additional tools for evaluation of neuritis in leprosy [3]. Martinoli et al. [3] examined the median, ulnar and posterior tibial nerve in 23 leprosy patients (58 nerves) both with sonography and MRI. Based on the sonographic (or MR) imaging appearance, a nerve could be classified as normal (group I), enlarged with fascicular abnormalities (group II) or having no fascicular structure at all (group III). The nerves in group II were thicker than in group III. The nerve swelling found in group II was gradual and fusiform, and typically occurred proximal to osteofibrous tunnels. However, their main finding was that nerves which showed a reversal reaction towards a more intense immune response had a hypervascular pattern demonstrated by Doppler studies (or by a marked T2 intensity and increased gadolinium enhancement on MR). That study had some limitations. It took the authors 3.5 years to examine 23 consecutive patients, but more importantly, the patients had mean disease duration of 15 years compared to 24.7 months in our study. Moreover, only affected clinical nerves were examined by sonography or MRI, while we examined the MN, UN, LP and PT nerves systematically in all leprosy patients. Finally, they did not compare the imaging results with clinical findings.

As expected, we also found that nerves are often enlarged in leprosy patients, especially in patients with a type 1 or 2 reaction. One of the three key signs of leprosy is the presence of enlarged nerves. Ascertaining the presence of enlarged nerves can be difficult [1], and for some nerves this is impossible because of their location. Additionally, it is impossible to assess the length of nerve abnormality by palpation. There is considerable inter-observer variability in assessing the presence of enlarged nerves by palpation [1],[13]. In contrast, US is a very precise assessment method as shown in a study of cadavers [14]. Furthermore, inter-observer agreement between sonographic measurements is excellent [9]. Our study clearly indicates that the kappa between clinical palpation and assessment of nerve size by sonography is low and that (taking the earlier observations into account) clinical palpation to assess nerve enlargement is inferior to US. It has to be emphasized that palpation of nerves in our study was performed by very experienced clinicians. We conclude that clinical examination of enlarged nerves is subjective and inaccurate, whereas sonography provides an objective measure of the nerve dimensions in addition to revealing structural changes over a longer length of the nerve.

Besides enlargement, nerves in leprosy patients exhibited varying degrees of structural abnormalities such as fusiform enlargement or loss of fascicles, edema and increased neural vascularity. This confirmed earlier findings [3]. Nerves that showed increased blood flow signals in the endo/perineurium belonged to patients with leprosy reactions, as Martolini et al. also demonstrated. As compared to the nerve size or echotexture, the above feature discriminates leprosy reactions from non-reaction leprosy. However, in the present study, sonography was unable to discriminate between reversal and ENL reactions, although multiple nerve involvement was seen more often in ENL reactions. Interestingly, increased blood flow was seen in contralateral nerves and in multiple nerves distant to the affected dermal lesion, indicating that inflammation in the nerve may be more wide spread than suggested by the dermal lesions. We found that the more enlarged the nerve, the more often CD flow signals were present. Possibly, an increased blood flow signal in the nerve is the first sign of possible nerve damage. For example in 12 of 23 UN, CD flow signals were found while no sensory or motor nerve impairment was observed. Moreover, some nerves (8%) have increased CD blood flow signals without being enlarged. A prospective study is ongoing to assess the presence of increased CD flow signals on the development of nerve enlargement and clinical nerve impairment.

Our observations confirm the findings that nerve enlargements extend far proximal to the compression sites of the UN and MN [3], occasionally with a nerve length abnormality of 22 cm. However, our preliminary data indicate that the maximum nerve enlargement is not just proximal to the possible compression sites, but for the MN approximately 4 cm from the proximal carpal tunnel inlet and for the UN 4–6 cm above the sulcus. This suggests that for these sites the temperature of the nerves could be lowest and more prone to infection by *Mycobacteriun leprae*, which is thought to favor lower body temperatures [15],[16]. These findings need confirmation, since it may indicate that nerve release surgery at entrapment sites is based on inadequate assumptions.

The increased neural vascularity taken together with interfascicular edema may reflect immune-mediated inflammation known to occur during leprosy reactions [17],[18]. Though in general such nerves showed both abnormal echotexture and higher CSA, the exception of 7 nerves with normal CSA and echotexture leads us to believe that increased vascularity may be a better marker of acute neuritis associated with leprosy reactions. Increased CSA and abnormal echotexture may reflect chronic, long term effects of leprosy. We believe that using sonography, these processes and progressive nerve damage can be followed and a follow-up study is ongoing to assess the long-term value of US in leprosy. The anti-reaction treatment is discontinued upon clinical amelioration and some patients develop repeated leprosy reactions even after a full course of treatment. In such cases, color Doppler imaging may assist in judging the return to normalcy following neuritis and the time that anti-reaction treatment is needed.

## Supporting Information

Video S1 Video clip of endoneural flow by color Doppler of peripheral nerves in BT leprosy patient undergoing type 1 reaction. Legend: Endoneural flow in right ulnar nerve (CSA = 51 mm^2^) - longitudinal view.
(5.04 MB AVI)

Alternative Language Abstract S1 Translation of the abstract into Dutch by Leo H. Visser
(0.02 MB DOC)

Alternative Language Abstract S2 Translation of the abstract into German by Joseph Bohm
(0.03 MB DOC)

## References

[1] Wilder-Smith EP, Van Brakel WH (2008). Nerve damage in leprosy and its management.. Nat Clin Pract Neurol.

[2] Ridley DS, Jopling WH (1966). Classification of leprosy according to immunity. A five-group system.. Int J Lepr Other Mycobact Dis.

[3] Martinoli C, Derchi LE, Bertolotto M, Gandolfo N, Bianchi S (2000). US and MR imaging of peripheral nerves in leprosy.. Skeletal Radiol.

[4] WHO seventh expert committee (2009). report on leprosy (1998): 7th Technical report series..

[5] Brandsma W (1981). Basic nerve function assessment in leprosy patients.. Lepr Rev.

[6] Weinstein S (1993). Fifty years of somatosensory research: from the Semmes-Weinstein monofilaments to the Weinstein Enhanced Sensory Test.. J Hand Ther.

[7] Jain S, Muzzafarullah S, Peri S, Ellanti R, Moorthy K (2008). Lower touch sensibility in the extremities of healthy Indians: further deterioration with age.. J Peripher Nerv Syst.

[8] Beekman R, Visser LH (2004). High-resolution sonography of the peripheral nervous system – a review of the literature.. Eur J Neurol.

[9] Beekman R, Schoemaker MC, Van Der Plas JP, van den Berg LH, Franssen H (2004). Diagnostic value of high-resolution sonography in ulnar neuropathy at the elbow.. Neurology.

[10] Beekman R, van den Berg LH, Franssen H, Visser LH, van Asseldonk JT (2005). Ultrasonography shows extensive nerve enlargements in multifocal motor neuropathy.. Neurology.

[11] Tagliafico A, Resmini E, Nizzo R, Bianchi F, Minuto F (2008). Ultrasound measurement of median and ulnar nerve cross-sectional area in acromegaly.. J Clin Endocrinol Metab.

[12] Visser LH (2006). High-resolution sonography of the common peroneal nerve: detection of intraneural ganglia.. Neurology.

[13] Chen S, Wang Q, Chu T, Zheng M (2006). Inter-observer reliability in assessment of sensation of skin lesion and enlargement of peripheral nerves in leprosy patients.. Lepr Rev.

[14] Kamolz LP, Schrogendorfer KF, Rab M, Girsch W, Gruber H (2001). The precision of ultrasound imaging and its relevance for carpal tunnel syndrome.. Surg Radiol Anat.

[15] Dastur DK, Pandya SS, Antia NH (1970). Nerves in the arm in leprosy. 2. Pathology, pathogenesis and clinical correlations.. Int J Lepr Other Mycobact Dis.

[16] Sabin TD, Hackett ER, Brand PW (1974). Temperatures along the course of certain nerves often affected in lepromatous leprosy.. Int J Lepr Other Mycobact Dis.

[17] Modlin RL, Melancon-Kaplan J, Young SM, Pirmez C, Kino H (1988). Learning from lesions: patterns of tissue inflammation in leprosy.. Proc Natl Acad Sci U S A.

[18] Sreenivasan P, Misra RS, Wilfred D, Nath I (1998). Lepromatous leprosy patients show T helper 1-like cytokine profile with differential expression of interleukin-10 during type 1 and 2 reactions.. Immunology.

