# Supplementary material for: High-Resolution Sonography: A New Technique to Detect Nerve Damage in Leprosy
Source: PLoS Negl Trop Dis. 2009 Aug 11;3(8):e498. doi: 10.1371/journal.pntd.0000498 (PMC2716078; doi:10.1371/journal.pntd.0000498)
Supplement: Alternative Language Abstract S1 — Translation of the abstract into Dutch by Leo H. Visser (0.02 MB DOC) [file pntd.0000498.s002.doc]

**Abstract in Dutch**

**Samenvatting**

*Achtergrond*

Lepra is wereldwijd de meest voorkomende behandelbare aandoening van de perifere zenuwen, gekenmerkt door perioden met acute neuritis, hetgeen leidt tot functionele beschadiging van de armen en benen, ulcus vorming en stigmatiserende deformaties.

Aangezien de hoofdkenmerken van lepra zenuwverdikking en inflammatie zijn, hebben wij hoge resolutie echografie met kleuren Doppler gebruikt om zenuwverdikking en inflammatie aan te tonen.

Methoden en resultaten

Wij hebben bij 20 patiënten met lepra aan beide zijden de n. ulnaris (UN), n.medianus (MN), n. peroneus (PN) en n. tibialis posterior (TP) echografisch onderzocht en de echografische bevindingen vergeleken met de klinische bevindingen van deze patiënten en de echografische bevindingen bij 30 gezonde controles uit India.

De zenuwen waren bij de lepra patiënten significant dikker in vergelijking met de gezonde controles (p<0.001 voor alle zenuwen). Twee patiënten hadden geen zenuwverdikkingen en geen van beide patiënten had een type 1 of 2 reactie of klinische tekenen van neuritis. De kappawaarde voor klinische palpatie en zenuwverdikking bij echografie bedroeg 0.30 voor alle onderzochte zenuwen ( 0.32 voor de UN, 0.41 voor PN and 0.13 voor TP). Toegenomen vascularisatie in de zenuwen, vastgesteld door middel van kleuren Doppler, werd bij 39 van de 152 onderzochte zenuwen (26%) gevonden. Toegenomen vascularisatie werd in meerdere zenuwen bij 6 van de 12 patiënten met een type 1 reactie en bij 3 van de 4 patiënten met een type 2 gezien. Duidelijke significante correlaties werden gevonden tussen de klinische bevindingen (de mate van verdikking van de zenuwen, sensibiliteitsstoornissen en spierzwakte) en de echografische bevindingen van zenuw (mate van echo reflectiviteit, endoneurale vascularisatie en oppervlakte van de zenuwen) (allen, p<0.001).

Conclusies/belang van dit onderzoek

We concluderen dat bij patiënten met lepra klinisch onderzoek naar verdikte zenuwen subjectief en inaccuraat is. Echografie van de zenuwen daarentegen is een objectieve maat voor het vastleggen van zenuwbeschadiging bij patiënten met lepra door het vaststellen van verdikking van de zenuwen, waarbij tevens toegenomen vascularisatie en de mate van afwijkende echostructuur beoordeeld kunnen worden. Deze beschadigingen blijkt bij echografie groter en meer zenuwen te betreffen dan op basis van klinisch onderzoek vermoed wordt.

Translation Dr Leo H visser
