# Supplementary material for: High-Resolution Sonography: A New Technique to Detect Nerve Damage in Leprosy
Source: PLoS Negl Trop Dis. 2009 Aug 11;3(8):e498. doi: 10.1371/journal.pntd.0000498 (PMC2716078; doi:10.1371/journal.pntd.0000498)
Supplement: Alternative Language Abstract S2 — Translation of the abstract into German by Joseph Bohm (0.03 MB DOC) [file pntd.0000498.s003.doc]

# Zusammenfassung

## Hintergrund

Lepra ist die weltweit häufigste behandelbare Erkrankung der peripheren Nerven mit Episoden einer akuten Neuritis, die zu funktionellen Behinderungen der Gliedmaßen, zu Geschwüren und zu stigmatisierenden Deformitäten führt. Da die Nervenverdickung und die Entzündung Kennzeichen der Lepra sind, verwendeten wir die hochauflösende Sonographie und die Farbduplexsonographie (CD) um diese Veränderungen zu demonstrieren.

*Methode/ wichtigste Befunde*

Wir führten beidseitige Ultraschalluntersuchungen des N. ulnaris (NU), N. medianus (NM) , N. popliteus lateralis (PL ) und N. tibialis posterior (TP) bei 20 Leprapatienten durch und verglichen diese mit den klinischen Befunden der Betroffenen sowie mit den sonographischen Befunden von 30 gesunden Indern. Die Nerven waren signifikant dicker bei den Leprapatienten als bei den Kontrollpersonen ( p < 0,0001 für jeden Nerv). Die beiden Patienten ohne Nervenverdickung hatten keine Typ 1- oder Typ 2 - Reaktion oder Zeichen der Neuritis. Das Kappa für die klinische Palpation und für die Nervenverdickung betrug sonographisch 0,30 für alle untersuchten Nerven ( 0,32 für NU, 0,41 für TP und 0,13 für PL). Eine verstärkte neurale Vaskularisation fand sich im Farbduplex bei 39 von 152 untersuchten Nerven ( 26%). Verstärktes Farbduplex-Signal in mehreren Nerven wurde bei 6 von 12 Patienten mit Typ 1-Reaktion und bei 3 von 4 Patienten mit Typ 2-Reaktion beobachtet. Eine signifikante Korrelation wurde zwischen den klinischen Parametern der Nervenverdickung, dem sensorischen Verlust, der motorischen Schwäche und den Ultraschall-Abnormitäten der Echotextur, des endoneuralen Flusses und der Querschnittsfläche beobachtet (p<0.001) .

*Schlussfolgerungen/ Bedeutung*

Wir schlussfolgern, dass die klinische Untersuchung einer Nervenverdickung subjektiv und ungenau ist , wohingegen die Sonographie ein objektives Maß der Nervenschädigung liefert durch den Nachweis der verstärkten Vaskularisation, der veränderten Echotextur und der Verdickung. Die Schädigungen sind sonographisch umfangreicher und betreffen mehr Nerven als klinisch erwartet.

Translation by Joseph Bohm
